# Silent Free Fall at Disease Onset: A Perspective on Therapeutics for Progressive Multiple Sclerosis

**DOI:** 10.3389/fneur.2018.00973

**Published:** 2018-11-27

**Authors:** Patrizia LoPresti

**Affiliations:** Department of Psychology, University of Illinois at Chicago, Chicago, IL, United States

**Keywords:** oligodendrocytes, multiple sclerosis, CNS repair, neuroprotection, myelin repair, inflammation, disability prevention

## Abstract

Central nervous system (CNS) degeneration occurs during multiple sclerosis (MS) following several years of reversible autoimmune demyelination. Progressive CNS degeneration appears later during the course of relapsing-remitting MS (RRMS), although it starts insidiously at disease onset. We propose that there is an early subclinical phase also for primary-progressive (PP) MS. Consensus exists that many different cell types are involved during disease onset. Furthermore, the response to the initial damage, which is specific for each individual, would result in distinct pathological pathways that add complexity to the disease and the mechanisms underlying progressive CNS degeneration. Progressive MS is classified as either active or not active, as well as with or without progression. Different forms of progressive MS might reflect distinct or overlapping pathogenetic pathways. Disease mechanisms should be determined for each patient at diagnosis and the time of treatment. Until individualized and time-sensitive treatments that specifically target the molecular mechanisms of the progressive aspect of the disease are identified, combined therapies directed at anti-inflammation, regeneration, and neuroprotection are the most effective for preventing MS progression. This review presents selected therapeutics in support of the overall idea of a multidimensional therapy applied early in the disease. This approach could limit damage and increase CNS repair. By targeting several cellular populations (i.e., microglia, astrocytes, neurons, oligodendrocytes, and lymphocytes) and multiple pathological processes (e.g., inflammation, demyelination, synaptopathy, and excitatory/inhibitory imbalance) progressive MS could be attenuated. Early timing for such multidimensional therapy is proposed as the prerequisite for effectively halting progressive MS.

## Introduction

Each year, multiple sclerosis (MS) affects ~2.0 million people worldwide, resulting in ~20,000 deaths from this disease (1). MS is a central nervous system (CNS) degenerative disease with autoimmune demyelination and progressive CNS degeneration. Accurate disease classification is necessary for an effective understanding and treatment of MS, with emphasis on the progressive CNS degeneration component, which so far has eluded definitive characterization (1, 2). In 1996, MS was classified into various disease types based on the clinical phenotypes only (3). In 2013, the International Advisory Committee on Clinical Trials of MS proposed descriptors of the disease that included clinical relapse rate and imaging findings for disease activity, combined with disease progression (4). More recently, Lublin provided new MS phenotypic classification. Progressive MS includes active and inactive progressive MS with and without progression (5). Activity is defined by the presence of clinical relapses and/or new/enlarging lesions detected by magnetic resonance imaging, whereas progression is defined by increased disability within a definite period (~1 year). In addition, MS forms include relapsing-remitting disease and the clinically isolated syndrome. These two disease groups can also be not active and active.

The progressive degenerative component of the disease might always be present, albeit subclinically at disease onset. The most important objectives for future research in progressive MS are to determine the rate of progressive decline at the very beginning of the disease and to identify factors that can be pharmacologically targeted. The rate of progressive decline might be determined by differences in the degree and activation of inflammatory cells, as well as the CNS sites affected by inflammation.

It is possible that subclinical deterioration is present in all MS variants, starting at the disease onset. Consistent with this possibility, we found both remitting and progressive processes in an animal model of relapsing-remitting MS (RRMS) (6). We found that RR-experimental autoimmune encephalomyelitis (RR-EAE) mice had reversible motor impairment and progressive memory decline during the first 30 days post-immunization (6). We propose that drugs potentially effective for progressive MS could be tested for their ability to significantly alter the rate of memory decline in RR-EAE mice. Should a selective pharmacological approach significantly alter the rate of memory decline in RR-EAE mice, such drugs could be tested for progressive MS. Consistent with our report in the animal model of MS, clinicians have previously reported patients with subclinical incremental cognitive deterioration, i.e., ongoing CNS degenerative function clinically undetectable for a definite period during the disease (7, 8). Indeed, previous studies found that clinically silent T2 lesions affect cognition in early RRMS (9, 10). With regard to progressive MS, clinical studies have also shown that primary-progressive (PP) MS patients have an impaired ability to use newly learned information (11), cognitive decline over time (7), and lesions in clinically silent CNS regions (12, 13). Notably, mild cognitive impairment is considered to precede neurodegeneration and dementia (14).

Any treatment that targets early pathogenetic mechanisms would not be able to work over time, because early disease mechanisms might evolve along separate pathways, and effective treatments at later stages would require targeting the mechanisms underlying progression, but these remain to be elucidated. Until mechanisms explaining disease progression are identified, therapies applied at the earliest time of disease and directed toward anti-inflammation, regeneration, and neuroprotection are the best means to prevent the most debilitating clinical outcomes of progressive MS and poor quality of life. This review presents selected therapeutics in support of the overall idea of a multidimensional therapy applied early in the disease.

Pathological differences between SPMS and PPMS reportedly indicate separate entities (12, 15). However, whether the heterogeneous pathological patterns found in a biopsy are also present at the onset of the disease remain unknown. Before the onset of either clinically evident PPMS or SPMS, ongoing subclinical dysfunction might develop via various pathogenetic mechanisms that eventually manifest as distinct entities in biopsies of RRMS and PPMS patients. MS is a multifaceted disease at onset and its complexity increases over time; that is, the disease advances along multiple pathways specific for each patient. An important question is whether early in disease progression, the inflammatory response differs in RR vs. progressive MS. Anti-inflammatory treatment is not effective in progressive MS that is already clinically apparent; however, a selected inflammatory component of the disease might be present during the very early subclinical phase. We propose that any pharmacological treatment of SPMS would have to start at the onset (of what appears as RRMS) for an effective chance of stopping the onset of clinically evident progressive MS. In contrast, therapeutic intervention later would have to target the mechanisms of progressive CNS degeneration.

Factors that have been postulated to directly affect the progressive decline of axonal function and neurodegeneration include microglia activation, oxidative stress, and mitochondria deficits (16). These dynamics are in place early during the disease and should be targeted at the very onset. In addition, iron overload reportedly plays a role in neurodegeneration, but perhaps at a later stage of the disease (17). Calabrese et al. (18) reported cortical lesions and atrophy associated with cognitive impairment in RRMS patients. Cortical lesions are involved in some aspects of cognitive deficits, but future studies should determine whether cortical lesions could be the cause of the progressive nature of the CNS decline. Kutzelnigg and Lassmann (19) reported MS lesions at cerebral cortex sites. Such lesions, associated with the progressive phase, are both inflammatory and demyelinating. First, chronic destruction of myelin caused by activated microglia at these cortical sites might indeed promote progressive cognitive losses because myelin debris alters long-term potentiation (LTP) (20). Second, antibodies against myelin basic protein could also have a role since they are associated with cognitive decline after strokes (21). Furthermore, the initial damage could be continuously amplified since T- or B-cells at these same sites produce soluble factors that diffuse into the cortical tissue and further destroy myelin. Another aspect that could be responsible for the cognitive decline is the newly formed myelin (which is generated following brain demyelination). However, the effects of brain demyelination/remyelination on LTP and cognitive functions require further investigation (22–25).

## Holding back progressive multiple sclerosis: an early challenge with promise

**Figure 1:**
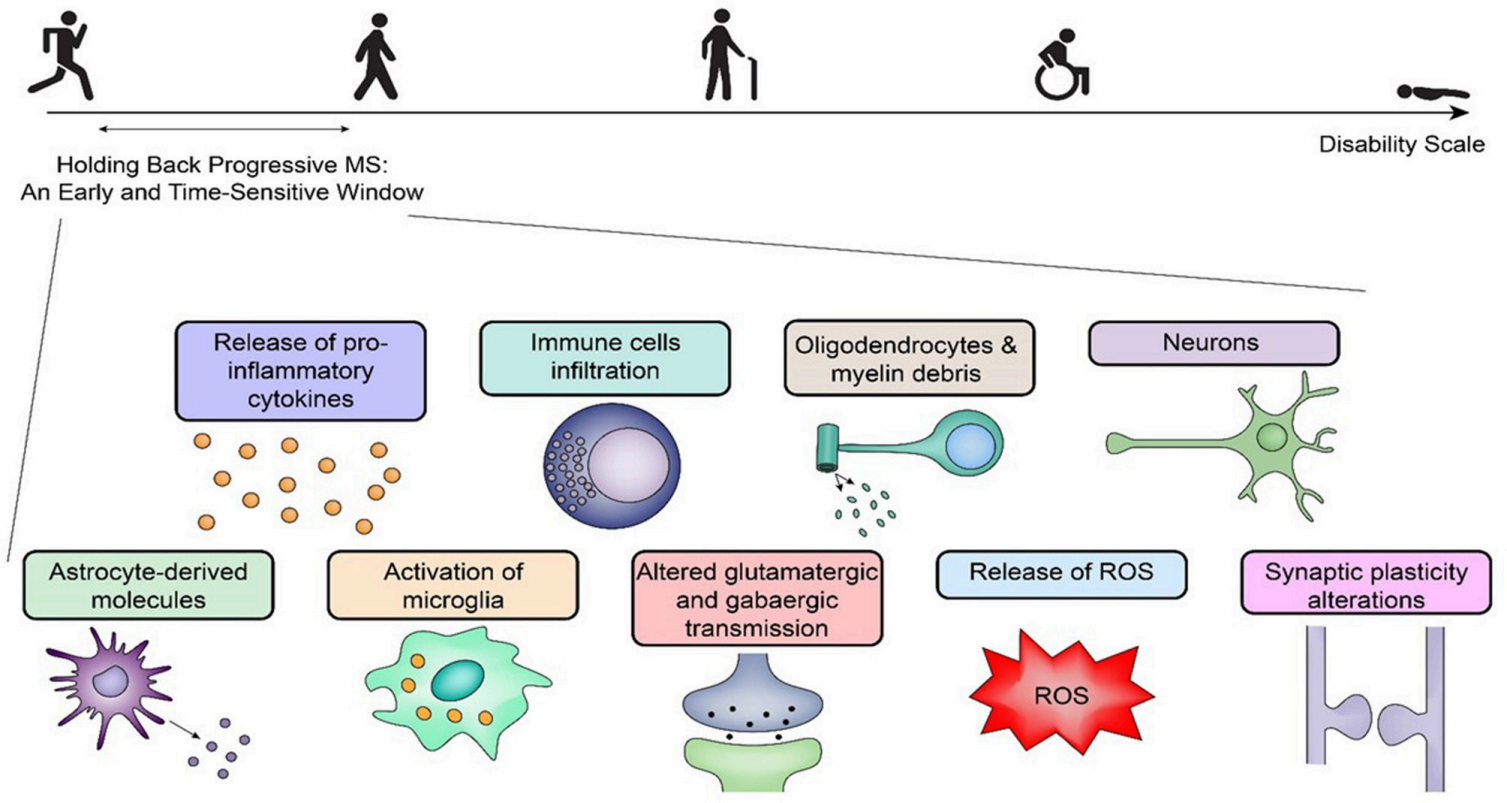
Holding back progressive multiple sclerosis: an early challenge with promise. Holding back progressive multiple sclerosis (MS) requires therapeutics within a time-sensitive window during the disease process. Early on, there are many pathogenetic pathways elicited by inflammation, and the response to the initial insult(s) is specific for each patient with distinct pathogenetic pathways in relapsing-remitting MS and primary-progressive MS. A selective pharmacological targeting requires an analysis of the mechanisms of the disease at the time of treatment. The figure outlines several early key players that can initiate progressive central nervous system degeneration via separate mechanisms, which poses additional challenges for any therapeutic agent to be effective later. ROS, reactive oxygen species.

Treatment of progressive MS would have to start at the very onset of the disease for all MS patients, because an early pharmacological approach would have a chance of halting degenerative processes that are clinically detectable only later during the disease (Figure 1). Effective treatments for progressive MS could be derived from approaches targeting inflammation and apoptosis in other diseases (26). In addition, therapeutics proven effective in progressive CNS degenerative diseases such as Parkinson's and Huntington's diseases might be tested in MS (27), because all MS types eventually become progressive CNS degenerative diseases. Inflammation is the most significant event the brain experiences following diverse insults (28, 29); it generates regions that locally damage the CNS area. During RRMS, several inflamed regions eventually become widespread CNS areas of degeneration during the final stages of SPMS. In contrast, during PPMS, the CNS degeneration remains more localized. Indeed, postmortem analyses of brains from patients with SPMS show diffuse degeneration, whereas those from patients with PPMS have more localized degeneration (30), although this finding could also be explained by disease duration.

## The many approaches for treating multiple sclerosis

### Targeting synaptic transmission

A finely tuned ratio of excitatory to inhibitory synaptic transmission supports neurogenesis and CNS health (31). In contrast, an imbalance initiates excitotoxic damage together with a pattern of CNS degeneration independent of inflammation (32). Several studies have shown that neurodegeneration can be caused by a synaptic transmission ratio that has been altered due to various factors present during CNS degenerative diseases (32, 33).

Overactivation of *N*-methyl-D-aspartate (NMDA) and α-amino-3-hydroxy-5-methyl-4-isoxazolepropionic acid (AMPA) receptors by the excitatory neurotransmitter glutamate causes neuronal damage; whereas beneficial effects are obtained by targeting these receptors in animal models of MS. Glutamate is the major excitatory neurotransmitter. Elevated glutamate concentrations have been found in MS lesions (34); an excess of glutamate leads to calcium increases and can be antagonized via AMPA or NMDA receptor blockers (34), with positive effects on axons.

EAE mice treated with pharmacological treatments that target the glutamatergic system have reduced disease activity (32, 35–38). The weak NMDA receptor antagonist, amantadine, improves the disease (32, 34), whereas riluzole (both a sodium channel blocker and a kainate and NMDA receptors antagonist) decreases inflammation, demyelination, and axonal damage (32, 35, 37). Clinical trials involving MS patients have shown that amantadine reduced the relapse rate in RRMS (32, 36), whereas riluzole reduced lesion evolution and axonal loss, with no positive effect on the formation of new lesions during PPMS (32, 37). However, riluzole treatment did not significantly reduce brain atrophy progression in early MS (32, 37). Modulation of synaptic transmission also presents challenges in MS patients. For example, the use of memantine that acts on the glutamatergic system by blocking NMDA receptors caused neurological impairment in MS patients, although the impairment is reversible (32, 39).

Regarding the inhibitory neurotransmitter gamma-aminobutyric acid (GABA), treatments that target the GABAergic system delay development of EAE disease and decrease EAE severity (32, 38). Treatment of MS patients with gabapentin (a GABA analog) ameliorated acquired nystagmus; whereas other similar drugs such as Vigabatrin and baclofen showed no effect in either RRMS or PPMS patients (32, 38). Overall, studies targeting the glutaminergic system have shown better results for MS patients than those targeting the GABAergic system, although modest differences might be missed in this later group of patients. In summary, in the context of neuroprotection, targeting LTP regulation might still provide valuable benefits, so this strategy should be further investigated.

Microglia are targeted to protect the ratio of excitatory to inhibitory synaptic transmission because these cells can affect the ratio in several ways. One mechanism involves microglia acting as a physical barrier to the inhibitory transmission (40). A second mechanism involves microglia directly pruning synapses via a complement-mediated mechanism, which has also been described during development and adult life (41). Although mitoxantrone induces microglial death when used *in vitro* (42), its use, which was approved for rapidly worsening RRMS and SPMS, was discontinued due to cardiotoxicity. Nevertheless, microglia could still be pharmacologically exploited to increase protection and reduce damage during progressive MS.

An earlier intervention targeting inflammation could also protect the ratio of excitatory to inhibitory synaptic transmission. Inflammatory cytokines released during the acute phase of the disease change the ratio of excitatory to inhibitory synaptic transmission (43, 44). In contrast, a massive loss of synapses in diffuse “synaptopathy” characterizes permanent functional deficits at a later stage of the disease (45, 46). Among inflammatory cytokines, Interleukin 1 (IL1) alters the ratio of excitatory to inhibitory synaptic transmission during inflammatory demyelination (44). Other factors secreted by T-cells such as nitric oxide (NO) and osteopontin have similar deleterious effects (47). Notably, osteopontin levels increase during progressive MS (48). However, whether an earlier intervention targeting downstream signaling pathways of IL1, NO, and osteopontin can protect the ratio of excitatory to inhibitory synaptic transmission and prevent functional CNS declines would require further testing. Furthermore, a recent study has shown that IL33 treatment inhibits cognitive dysfunction associated with experimental cerebral malaria, an inflammatory disease of the CNS (49). Thus, by learning the positive and negative effects of various cytokines, rationale approaches can be used to favor the protective cytokines. In this context, glibenclamide, an ATP-sensitive potassium channel blocker, should be tested for progressive MS, because it decreases the production of proinflammatory mediators (Tumor necrosis factor [TNF-α], IL-1β, and reactive oxygen species) and the accumulation of inflammatory cells (50).

### Targeting neurons

**Figure 2:**
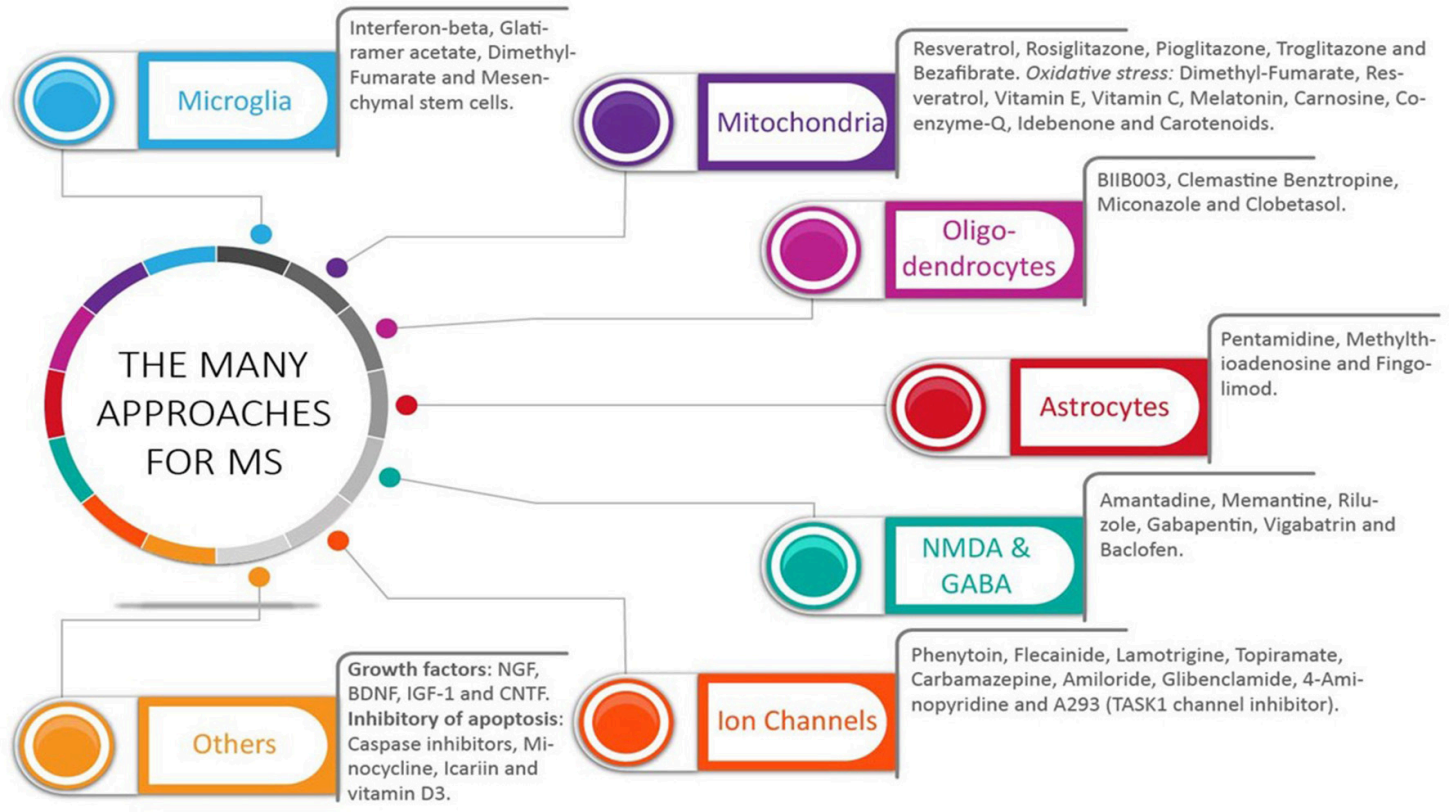
The many approaches for multiple sclerosis: what did we get. A wide range of pharmacological targets has been used to treat multiple sclerosis (MS). Selective drugs for each group are shown. The many targets have addressed the multifaceted aspect of this disease, which include both inflammation and central nervous system (CNS) cells, including neurons, oligodendrocytes, and astrocytes. Most studies have analyzed the effects of these drugs in clinically apparent relapsing-remitting MS and primary-progressive MS. Without an identification of the mechanisms in place during this disease, potential benefits could have been missed in those MS patients with ongoing subclinical CNS progressive degeneration.

Neurons are vulnerable during demyelinating-inflammatory diseases. First, demyelination changes sodium channel regulation and nerve conduction with downstream compensatory mechanisms involving calcium influx and changes in calcium homeostasis (51, 52). Second, inflammation changes axonal transport regulation (53, 54). Regarding drugs targeting sodium channels, those directed to voltage-gated sodium channels protect axons, reduce inflammation, and decrease disease severity (55). Amiloride, an inhibitor of sodium entry, has significant positive effects on neurodegeneration treatment as measured by magnetic resonance imaging (56); whereas 4-aminopyridine, a drug directed against potassium (K) channels, improves mobility (57). Furthermore, blocking potassium channels reduced axonal and neuronal degeneration in the Myelin Oligodendrocyte Glycoprotein (MOG35-55)-induced EAE MS model (58). Potassium channels are present on T-cells, so blocking two-pore domain weakly inward-rectifying K channel (TWIK)-related acid-sensitive K+ channel 1 (TASK1) also leads to less T-cell proliferation and reduced proinflammatory cytokines, which all have beneficial effects on neurons (59, 60) (Figure 2).

Regarding intracellular transport regulation, earlier interventions might be effective against neuronal functional deficits and neurodegeneration. A previous study reported axonal transport deficits at the onset of optic neuritis in EAE mice, whereas reduced levels of the axonal motor protein KIF5A (kinesin heavy chain isoform 5A) were found in MS patients (53, 54). Unfortunately, the mechanisms underlying alterations of axonal transport regulation have largely eluded our understanding, so rational approaches for correcting anomalies of axonal transport are not available.

In the context of neuroprotection, early targeting of inflammation also reduces proinflammatory molecules produced by macrophage/microglia. Such molecules are deleterious for the mitochondria, which provide energy for neurons (61, 62). Several potential compounds targeting the mitochondria have been identified (63) (Figure 2). Impairment of mitochondrial function and subsequent energy loss is a consequence of both reactive oxygen and nitrogen species, which are abundant in MS lesions (64). Indeed, oxidative stress has been identified to lead to progressive CNS degeneration in Parkinson's disease (65), and oxidative stress levels have been directly linked to the progression of MS (66). Early antioxidant therapy is believed to limit CNS progressive degeneration (66); several antioxidants are in preclinical or already phase 1 and 2 clinical trials for MS patients (Figure 2) (66). Benefits for neuroprotection are obtained via activation of the Nrf2-antioxidant response element-signaling pathway, as shown by using fumaric acid esters (Figure 2) (67). Antioxidant therapies should start at the earliest possible time to halt pathways underlying CNS progressive degeneration.

### Targeting oligodendrocytes and myelin

Improving myelin repair is expected to be neuroprotective (68, 69). During demyelinating diseases, demyelination in the spinal cord (70) causes a mobility defect that is remitting, due to remyelination and consequential functional recovery. In contrast, the consequences of demyelination/remyelination in the brain remain largely unknown, with emphasis on the consequences on LTP and cognitive functions (22). Nicaise et al. (71) showed that induced pluripotent stem-derived neural progenitor cells from PPMS patients had defective myelin repair. Thus, by increasing myelin repair, devastating progressive disability should be eased (72). Fingolimod (FTY720), the first US Food and Drug Administration (FDA)-approved oral medication for MS, increases neural stem cell survival and enhances their development into mature oligodendrocytes (OLGs) (73), with benefits for myelin repair. The water-soluble B vitamin biotin also has positive effects on myelination; it is in clinical trials for SPMS (74). In addition, several compounds have been shown to increase myelin. In this respect, antihistamines and muscarinic receptor antagonists are valuable, and selected compounds have been selected for clinical trials. Within this group, both Clemastine and GSK239512 led to improvement in functional assessments and lesions (75–80). Other compounds such as benztropine, which works as an anticholinergic, antihistamine, and dopamine reuptake inhibitor, improves myelin levels, but no clinical trials have been started (72). In addition, LINGO (leucine-rich repeat and immunoglobulin-like domain-containing protein 1) and semaphorin inhibit myelination (81–87). Antibodies to these two distinct sites have been developed. Clinical studies have presented various challenges. However, these approaches should be explored in more detail. Furthermore, a variety of compounds exerts positive impacts on myelination. These include remyelinating-promoting IgM (rHIgM22), a non-selective G protein-coupled receptor antagonist (Quetiapine), a dopamine 2 receptor antagonist (Domperidone), thyroid hormone-like compounds (Liothyronine sodium, a T3 thyroid hormone), estrogen receptor modulators, agonists for retinoic acid receptors (RXR-γ), glucocorticoid (clobetasol), kappa opioid receptor agonists (U-50488), adrenocorticotrophic hormone, and erythropoietin (72, 88–97).

A new therapeutic area for improving myelin repair should also target the cytoskeleton of OLGs. In particular, the tau protein in oligodendrocytes is a key player during myelination (98, 99), so focusing on oligodendrocyte tau may boost myelin repair and CNS functions (100, 101), which would limit progressive MS (Figure 2).

### Targeting microglia

Microglia represent an important pharmacological target for CNS degeneration. They can exert either protective or deleterious effects on CNS cells through separate mechanisms. For example, microglial-mediated innate immunity results in CNS degeneration during Alzheimer's disease (41, 102). In contrast, microglia can protect the CNS through M2-dependent muscarinic receptor actions. Consistent with this effect, widely used FDA-approved drugs for MS such as interferon beta and Glatiramer acetate, exert neuroprotection via an M2-dependent pathway (102) (Figure 2).

### Targeting astrocytes

Several drugs targeting astrocytes are now available (Figure 2). During CNS inflammation, astrocytes release cytokines, which are deleterious to neurons (103). Fingolimod may support neuroprotection by blocking astrocyte NO (103). Furthermore, astrocytes are known to decrease the deleterious effects of glutamate because they express glutamate transporter-1, whereas decreased glutamate transporter-1 activity (in astrocytes) occurs during several CNS degenerative diseases (104), which lessens the ability of these cells to buffer glutamate and its toxic effects. At the same time, during the progressive stage of MS, selected astrocytes express lactosylceramide (LacCer), which recruits inflammatory monocytes from the blood (105). Thus, therapeutics that modulate the expression of the glutamate transporter-1 and LacCer in astrocytes might inhibit progressive MS.

### Targeting trophic support and growth factors

Growth factors, which are essential for the health of CNS cells, support efficient intracellular transport in neurons and other CNS cells (106–110). Targeting nerve growth factor (NGF) has been proposed to induce neuroprotection in MS (108). Of interest, trophic factors such as NGF also affect brain inflammation. NGF switches the balance of T-helper cell type 1 and 2 cytokines within the CNS during EAE (109). Furthermore, brain-derived neurotrophic factor (BDNF) has been reported to increase upon Glatiramer acetate treatment during developmental myelination, with positive effects on myelination (106). BDNF also protects against neuropathology in a mouse model of Alzheimer's disease (110) (Figure 2).

### Targeting apoptosis

Inflammatory cells release several factors that induce apoptosis (47). Perforin and granzymes A + B, secreted largely by CD8+ cells, cause apoptosis; whereas TNF-α, Interferon-γ, Interleukin-17, and other cytokines secreted by CD4+ and CD8+ cells enhance glutamate excitotoxicity (47, 111). Protection from cell death could be obtained by using pharmacological inhibitors of first apoptosis signal receptor (FAS) and TNF-dependent apoptosis (112).

### Others

An aberrant immune response is believed to give rise to MS, both for the remitting and progressive forms (113). Thus, treatments aimed at recalibrating the dysfunctional immune response are urgently needed. Autologous Hematopoietic Stem Cell Transplant (AHSCT) is one such treatment (114). The change in regulatory T-cell populations achieved following AHSCT can certainly protect the MS patient if the treatment is initiated early in the disease. However, since the pathogenesis of MS disability and the mechanisms by which AHSCT exerts protection are largely unknown, caution is warranted to avoid overreaching expectations (114–119). An important therapeutic approach includes the use of mesenchymal stem cells, which have potent antioxidant effects and are neuroprotective *in vivo* (120). Neuroprotection can also be achieved by targeting multiple pathways known to regulate immunity, as the combined use of interferon beta and fumarate has shown (113, 121). Finally, inhibitors of protease-activated receptors and potassium voltage-gated channels can protect against granzyme B-induced neurotoxicity (122).

## A glimpse of hope for progressive multiple sclerosis

**Figure 3:**
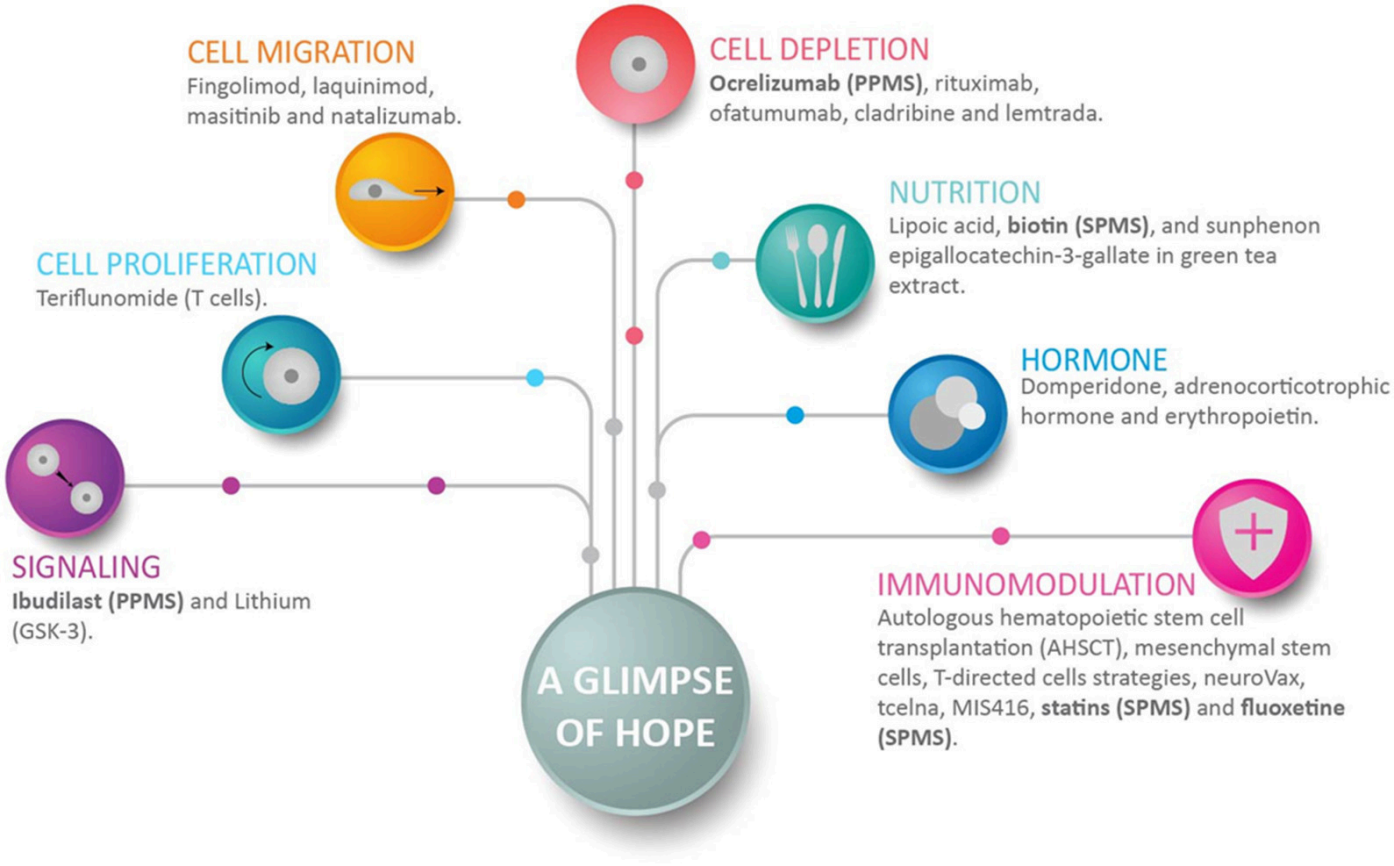
A glimpse of hope for progressive multiple sclerosis patients. This figure shows representative drugs for each group, and how anti-inflammation, neuroprotection, and myelin repair can be achieved. Anti-inflammation is achieved with drugs that inhibit cell proliferation, cell migration, and/or can deplete a selected cell population. Immunomodulation achieves several positive objectives that include neuroprotection. Drugs directed to signaling decrease neuroinflammation and promote neurogenesis. Of interest in the context of progressive multiple sclerosis (MS), recent drugs include ocrelizumab, which depletes B-cells; fluoxetine, which regulates astrocytes and microglia; and Ibudilast and biotin. Ibudilast suppresses proinflammatory cytokines, inhibits macrophage migration, upregulates the anti-inflammatory cytokine IL10, and increases neurotrophic factors (129). Biotin promotes axonal remyelination and reduces axonal hypoxia. Drugs enrolled in clinical studies of secondary-progressive MS and primary-progressive MS are shown in bold.

Although there is a consensus that by limiting the degree of inflammation the CNS benefits from a decrease of neuronal damage, additional approaches directed at neuronal signaling and in support of myelin repair are required to maximize the ability of the CNS to limit the damage and to increase repair (Figure 3). Anti-inflammation is achieved with drugs directed to cells involved in inflammation and immune responses. Such drugs inhibit cell proliferation, cell trafficking into the CNS, and/or can deplete a selected cell population. It is known that immunomodulation has benefits for neuroprotection. In the context of progressive MS, recent drugs include ocrelizumab that depletes B-cells (123–128), whereas Ibudilast suppresses proinflammatory cytokines, inhibits macrophage migration, upregulates the anti-inflammatory cytokine IL-10, and increases neurotrophic factors (129) (Figure 3). It should be taken into consideration that clinical studies might provide false negative for the potential benefits of selected therapeutics, since it is difficult to perfectly time the treatment with the disease. Perhaps an intervention during the subclinal phase, as proposed in this review, might provide better outcomes. For example, Fluoxetine by working as selective serotonin reuptake inhibitor, increases the amount of serotonin in the brain, and regulates astrocytes and microglia (130). However, the study by Mostert et al. (131) reported no benefits. Similarly, with regard to dietary supplements, biotin positively influences axonal remyelination and axonal hypoxia (74, 132). However, positive benefits were reported in some studies but not in others (133, 134). In addition, Rituximab, an anti-CD20 monoclonal antibody approved for non-Hodgkin lymphoma and rheumatoid arthritis, impacts the inflammatory aspect of the disease and RRMS activity, but its effect on PPMS progression appears to be marginal (135, 136). Finally, Teriflunomide is of potential interest. Teriflunomide primarily acts as an inhibitor of dihydroorotate-dehydrogenase (DHODH), a key mitochondrial enzyme involved in the de novo synthesis of pyrimidines in rapidly proliferating cells such as T- and B-lymphocytes, thereby diminishing the inflammatory response to auto-antigens (137). A comprehensive analysis of selected drugs presented in Figures 2, 3 are in previous reviews (32, 96, 138, 139).

In summary, while anti-inflammation, neuroprotection, and myelin repair constitute the combined approach of choice for progressive MS, early treatment is imperative to limit disability, and inhibit the mechanisms involved in progressive MS.

## Concluding remarks and future perspectives

Although this review offers general guidelines based on the available data, more research is required to select the drugs of choice. Overall, one or several targets at the very onset of the disease offer an effective treatment for progressive MS. We hypothesize that one or more of these early targets initiate a subclinical progressive demise of the CNS that later manifests as SPMS or much earlier as PPMS. An effective treatment must start at disease onset. In contrast, should it start when progressive MS becomes apparent, the critical window for intervention would be lost, and CNS degeneration would not be halted. Effective treatments for progressive MS must target disease onset, and they must be tailored to where the disease originates.

## Trends and outstanding questions

1. Progressive MS presents significant therapeutic challenges. MS is a multifaceted disease; its complexity increases over time.
2. A combination of drugs directed toward inflammation, neurons, and oligodendrocytes provides therapeutic options early during MS for the prevention of progressive MS.
3. Which is the cause of progressive MS?
4. Does the start of progressive MS occur during inflammation in the subclinical phase of this disease?
5. Until the mechanisms underlying progressive MS are identified, progressive MS is an early challenge that can be treated with agents that promote neuroprotection and myelin repair, and inhibit inflammation.
6. The time of treatment is critically important in limiting the progression of the multifaceted pathways of this disease.

## Author contributions

The author confirms being the sole contributor of this work and has approved it for publication.

## Conflict of interest statement

The author declares that the research was conducted in the absence of any commercial or financial relationships that could be construed as a potential conflict of interest.
